# Emergence of vaccine-derived poliovirus strains from the novel oral polio vaccine in the Central African Republic

**DOI:** 10.1128/mbio.00669-26

**Published:** 2026-04-23

**Authors:** Joël W. Doté, Marie-Line Joffret, Arthur Mazitchi, Dimitra Klapsa, Georges Kouatcho, Ernest Kalthan, Nolwenn Jouvenet, Javier Martin, Ionela Gouandjika-Vasilache, Maël Bessaud

**Affiliations:** 1 Institut Pasteur de Bangui–Laboratoire des virus entériques/rougeole, Bangui, Central African Republic; 2 Institut Pasteur–Université de Paris Cité–CNRS UMR 3569–Virus sensing and signaling Unit, Paris, France; 3 Laboratoire associé au Centre national de référence pour les entérovirus & paréchovirus, Paris, France; 4 Medicines and Healthcare products Regulatory Agency (MHRA), South Mimms, United Kingdom; 5 WHO office in Central African Republic, Bangui, Central African Republic; 6 Direction de le surveillance épidémiologique–Ministère de la santé et de la population, Bangui, Central African Republic; Georgia Institute of Technology, Atlanta, Georgia, USA

**Keywords:** poliovirus, novel oral polio vaccine, recombination, poliomyelitis, vaccine-derived poliovirus

## Abstract

The Central African Republic (CAR) is a high-risk country for poliomyelitis outbreaks because of difficulties in closing immunity gaps due to the lack of resources, logistics issues, and insecurity. The country experienced multiple poliomyelitis cases since 2019. A novel type 2 oral polio vaccine (nOPV2) engineered to lower the risk of reversion of the vaccine strain was deployed in the CAR in 2022–2023 to counter active serotype 2 poliovirus transmission. This study reports routine poliovirus surveillance conducted from November 2021 through October 2023 in the CAR. Polioviruses isolated from stools and wastewater were genetically characterized based on the VP1-encoding region that was sequenced by Sanger technique. Whole-genome sequencing was conducted on all nOPV2-derived isolates and on a subset of Sabin-2-derived isolates by using Illumina technology. Fifty-four vaccine-derived poliovirus type 2 isolates were identified, belonging to eight distinct emergences: four emergences were derived from the historical vaccine strain and four from the nOPV2. The nOPV2 derivatives that had kept the original 5′ untranslated region (5′UTR) did not display any reversions in the modified domain V, which is the primary attenuation site. Nonetheless, some nOPV2 derivatives had lost this domain through recombination with members of species *Enterovirus coxsackiepol* (EV-C). Our analyses demonstrate that recombination took place shortly after the vaccine rollout. The data demonstrate that nOPV2’s genetic stabilization curtails genetic drift-driven reversion but does not eliminate recombination. This underscores the need for comprehensive EV-C surveillance alongside poliovirus monitoring, especially in Sub-Saharan Africa where they are relatively abundant.

**IMPORTANCE:**

The novel oral polio vaccine of serotype 2 (nOPV2) was engineered to prevent the emergence of revertant polio vaccine strains, which caused numerous outbreaks in the 2000s and 2010. Since 2021, it has been used in many countries, especially in Sub-Saharan Africa, to contain poliomyelitis outbreaks. In 2023, double recombinant nOPV2-derived isolates that had lost all the attenuation determinants were detected in the Central African Republic (CAR). Several of these were the first nOPV2 revertant isolates ever reported to the Global Polio Eradication Initiative, thereby demonstrating that the engineered modifications in nOPV2 do not entirely prevent reversion via recombination with non-polio enteroviruses of species *Enterovirus coxsackiepol* (EV-Cs). This study corroborates the increased genetic stability of nOPV2 compared to the historical vaccine strain but demonstrates that reversion through recombination with non-polio EV-Cs remains possible. This underscores the need for comprehensive EV-C surveillance alongside poliovirus monitoring, especially in Sub-Saharan Africa where EV-Cs are particularly abundant. In particular, assessing EV-C potential seasonal circulation patterns could inform the design of vaccination strategies tailored to minimize the risk of recombination between nOPV2 and non-polio EV-Cs.

## INTRODUCTION

Launched in 1988, the Global Polio Eradication Initiative (GPEI) has managed to drastically reduce the global number of poliomyelitis cases and to eradicate wild strains of two of the three poliovirus serotypes. The GPEI is currently facing two challenges: (i) eradicating the last wild strains of poliovirus that still circulate in Pakistan and Afghanistan; (ii) preventing the emergence of revertant vaccine-derived polioviruses (VDPVs) that can induce poliomyelitis outbreaks. VDPVs emerge when live-attenuated strains comprising the oral polio vaccine (OPV) circulate for prolonged periods in populations with sub-optimal vaccine coverage and thus lose the attenuation determinants through genetic drift or recombination with non-polio enteroviruses of species *Enterovirus coxsackiepol* (EV-Cs). To date, the vast majority of VDPVs involved in outbreaks were derived from the vaccine strain of serotype 2 (Sabin 2) (1), which is the least genetically stable of the three vaccine strains selected in the 1950s. Many countries experienced VDPV2 outbreaks in the 2000s and 2010s (1). This situation was particularly worrisome because wild-type serotype 2 poliovirus strains were considered eradicated, with the last detection of a wild strain dating back to 1999. In order to prevent further VDPV2 outbreaks, the historic trivalent OPV was scheduled for replacement by a bivalent formulation containing only the vaccine strains of serotypes 1 and 3. The synchronized global switch was implemented in April 2016. Contrary to expectations, the strategy was followed by a marked increase in VDPV2 emergences during 2019 and 2020, especially in Sub-Saharan African countries (2). To contain these epidemics, the type-2 vaccine strain had to be re-deployed in a monovalent OPV, placing the program in a vicious circle in which doses administered to respond to one outbreak could seed the next (3). Breaking this cycle required the development of a novel type 2 oral polio vaccine (nOPV2). The nOPV2 has been engineered by modifying the genome of the historical Sabin 2 vaccine strain (4). The principal modification was the stabilization of the RNA stem-loop structure in the 5′ untranslated region (5′ UTR) known as domain V, which is the major determinant of Sabin 2 attenuation. To prevent the loss of this stabilized domain V through recombination, the essential cis-replication element (cre) located in the nonstructural region of the genome was disrupted (mutated cre) and relocated to the 5′ UTR (cre5). After its immunogenicity and genetic stability had been assessed in clinical trials, nOPV2 was granted World Health Organization’s Emergency Use Listing. Its rollout began in March 2021 in response to the numerous VDPV2 outbreaks that have arisen since the 2016 global switch. To date, more than two billion doses of nOPV2 have been administered in over 40 countries (5).

The Central African Republic (CAR) is a high-risk country for poliomyelitis outbreaks (6) that experienced multiple emergences of VDPV2 in 2019–2020 because of immunity gaps (7, 8). Routine polio immunization in the CAR relies on bivalent OPV with one dose at birth and three subsequent doses at 6, 10, and 14 weeks. Routine environmental surveillance is conducted through twice-monthly sampling of wastewater collected in Bangui, the capital city, and a few other towns. This study reports the results of polio surveillance activities conducted from 2021 through 2023 in the country. From Nov 2021, VDPV2s of different emergence groups were detected in the CAR. To stop their transmission, national vaccination campaigns were conducted with nOPV2. The aim of this study was to genetically characterize any poliovirus isolates detected by the routine surveillance to track their origin and determine whether the modifications engineered in the new vaccine strain abolish its ability to revert. The surveillance reported herein detected the emergence of nOPV2-derived VDPVs that lost the attenuation determinants by recombining both upstream and downstream the capsid-encoding genomic region with nonpolio EV-Cs. Some of these isolates were the very first nOPV2 revertant strains ever reported to the Global Polio Eradication Initiative.

## MATERIALS AND METHODS

### Sample collection and processing

This study covers the analysis of stools and wastewater collected from November 2021 through October 2023 in the CAR in the framework of national poliovirus surveillance. Stools were obtained from children with acute flaccid paralysis (AFP) and family or community contacts. No data were available regarding the race/ethnicity and the gender of the people from which the samples were collected. Wastewater was collected from collection sites sampled twice a month. Initially, thirteen sites were routinely sampled: four in Bangui, one in Berbérati, one in Bossangoa, one in Bouar, two in Bambari, one in Bangassou, one in Batangafo, one in Ndélé, and one in Alindao (Fig. S1). In 2022, two sites were closed because of low positivity rates (one in Berbérati and one in Bambari), while one was open in Bouar.

All stool and wastewater samples were processed by the Institut Pasteur de Bangui following the Global Polio Lab Network (GPLN) standard procedures for the detection of poliovirus by virus isolation in cell cultures (RD and L20B cell lines) and molecular intratypic differentiation (9). All supernatants positive for poliovirus of type 2 were spotted on FTA cards and sent to the polio Global Specialized Lab hosted by the Institut Pasteur, Paris, for sequencing.

### Genetic characterization of poliovirus isolates and phylogenetic analysis

All poliovirus type 2 isolates were characterized by Sanger sequencing of the VP1-encoding region (10) to discriminate Sabin-like isolates (<6 nt differences compared to Sabin 2) from VDPVs (≥6 nt differences). Nucleotide (nt) sequences were aligned with CLC Main Workbench (Qiagen, Venlo, Netherlands). The VDPV evolutionary history was inferred by using the maximum likelihood method and Tamura-Nei model with Mega X (11).

Whole-genome amplification of VDPV2s was performed with RT-PCR primers producing two overlapping amplicons (12). This protocol is not suitable for amplifying nonrecombinant nOPV2 derivatives since one of the primers targets the cre sequence that is modified in the nOPV2. Therefore, the genome of nOPV2 isolates was amplified using a tiled-PCR approach previously described (13). The sequencing libraries were created using 1 ng of DNA with the Nextera XT DNA Library Preparation Kit in a SureCycler 8800 thermocycler (Agilent). Following purification on AMPure beads (Beckman), the libraries were controlled using the High-Sensitivity D1000 assay (Agilent) on a TapeStation 2200. The products were sequenced by paired-end 150-bp *Illumina* sequencing using Illumina NextSeq HiSeq. All kits were used following the manufacturer’s instructions. At least one million reads were generated for each virus, ensuring a sequencing depth higher than 1,000.

Contigs were generated by *de novo* assembly with CLC Genomics Workbench (Qiagen, Venlo, Netherlands) using the following parameters: Update contigs = Yes, Automatic bubble size = Yes, Automatic word size = Yes, Perform scaffolding = Yes, Auto-detect paired distances = Yes, Mismatch cost = 2, Insertion cost = 3, Deletion cost = 3, Length fraction = 0.9, Similarity fraction = 0.95, and minimum contig length = 1,000 nt.

Similarity plots were drawn with the SimPlot software (14) using the Hamming distance method and standard parameters: 200-nt-wide sliding window and step size of 20 nt. Recombination breakpoints were located by visual inspection of pairwise alignments of VDPV sequences, with the nOPV2 sequence used as the reference.

## RESULTS

### Genetic characterization of poliovirus type 2 isolates based on the VP1-encoding region

**Fig 1:**
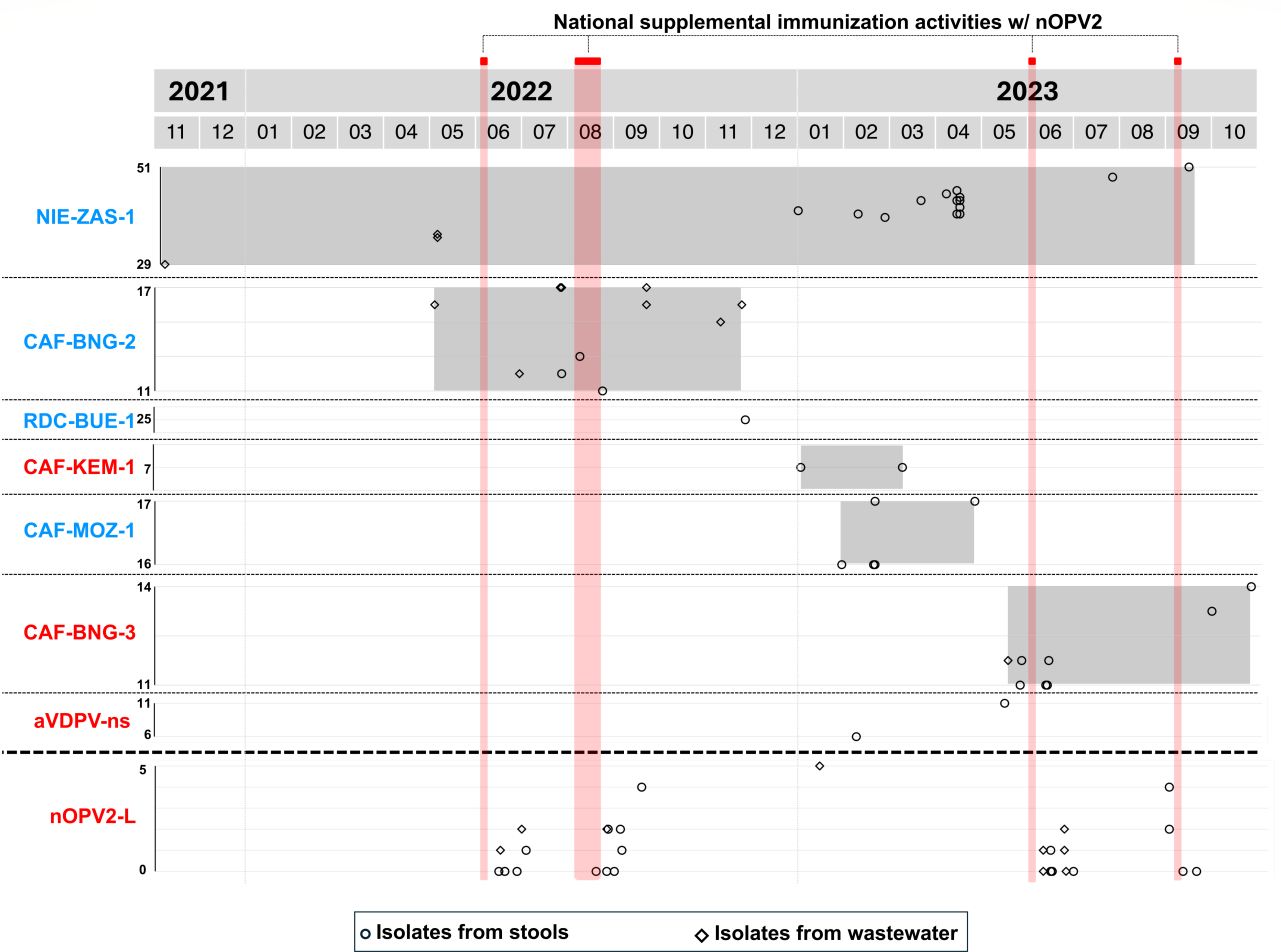
Timeline of poliovirus detection. Each dot represents a poliovirus isolate plotted according to the date of sampling (*x* axis) and the number of nucleotide differences in the VP1 compared to Sabin 2 (*y* axis). Sabin 2 derivatives are given in blue, and nOPV2 derivatives are given in red.

**Fig 2:**
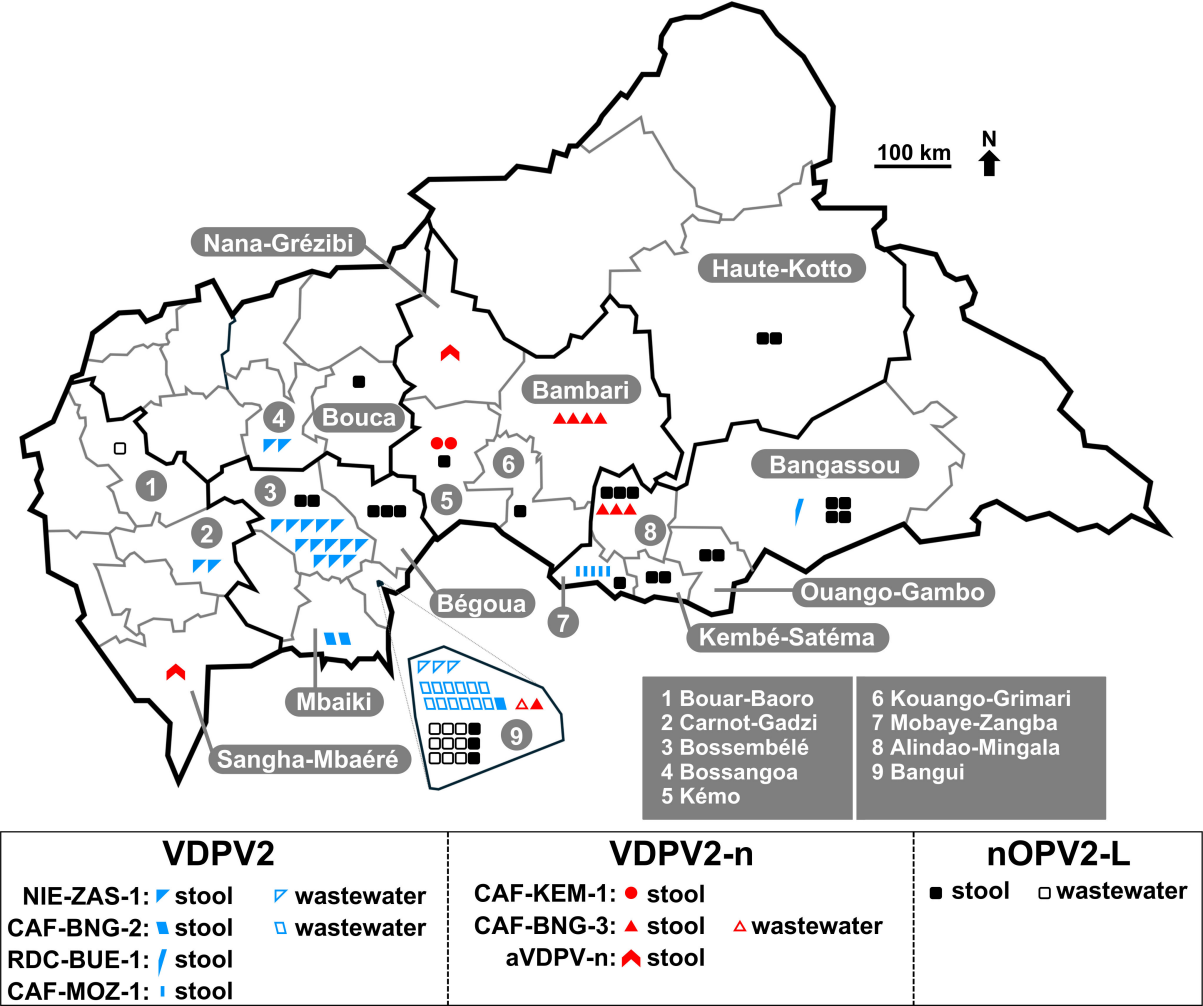
Map of the Central African Republic with health districts. Poliovirus isolates are plotted depending on their respective category (VDPV2, VDPV2-n, or nOPV2-L), emergence group, and source (stool or wastewater).

**Fig 3:**
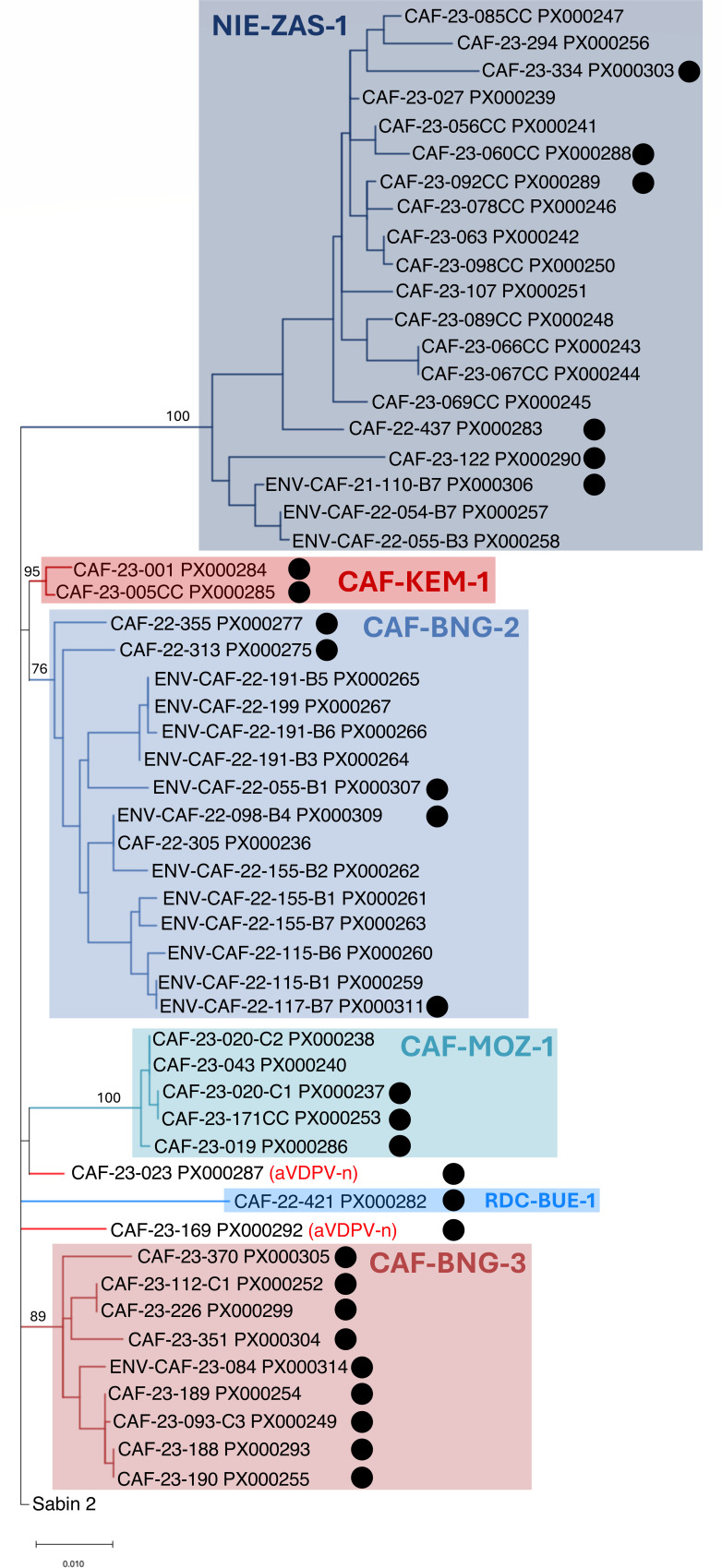
Phylogenetic tree based on the VP1-encoding nucleotide sequence. The evolutionary history was inferred by using the maximum likelihood method and Tamura-Nei model. VDPV2s are given in blue, and VDPV2-ns are given in red. Percent bootstrap values (1,000 replicates) of the main nodes are indicated. Full circles indicate isolates subjected to whole-genome sequencing.

While no poliovirus of serotype 2 had been detected in the CAR since November 2020, a VDPV2 was detected in a sewage sample collected in Bangui on 3 November 2021. It featured 29 nt differences in the VP1 compared to Sabin 2. The VP1 sequence of each newly identified VDPV is compared with those stored in the GPLN database to determine whether it is genetically linked to VDPVs sampled elsewhere. This analysis depends on identifying nt changes that are shared among VDPVs, relative to the vaccine strain (15). The new VDPV2 isolated in the CAR was found to be genetically linked to circulating VDPV2 isolates belonging to the emergence group NIE-ZAS-1, first detected in Nigeria in July 2020 (16). VDPV2s of the same emergence group were subsequently detected in two sewage samples collected in May 2022 in Bangui (Fig. 1). Concomitantly, another emergence with no genetic links to previous VDPV2s was detected in Bangui in May 2022 and named CAF-BNG-2 (Fig. 1 to 3). To respond to the active circulation of VDPV2s, national supplemental immunization activities (SIAs) were conducted with the nOPV2 in June and August 2022. It was the first time this vaccine was used in the CAR. The vaccine was administered to a population exceeding the target size (Table S1), but independent monitoring results were not available to the authors. Despite this response, isolates belonging to both NIE-ZAS-1 and CAF-BNG-2 emergences were being detected: NIE-ZAS-1 isolates were detected in three districts (Bossangoa, Bossembélé, and Carnot-Gadzi) from January 2023 through April 2023, while CAF-BNG-2 isolates were detected in Bangui and in Mbaïki district until November 2022. A VDPV2 detected in the Bangassou district, which borders the Democratic Republic of Congo, was genetically linked to isolates of the emergence group RDC-BUE-1, which was then circulating in the Democratic Republic of Congo (16); no other member of this emergence group was detected subsequently in the CAR. In 2023, three new emergences were detected for a few months each (Fig. 1 to 3): CAF-KEM-1 (two isolates in Kémo district), CAF-MOZ-1 (five isolates in Mobaye-Zangba district), and CAF-BNG-3 (nine isolates in Bangui and in Bambari and Alindao-Mingala districts). At the same period, NIE-ZAS-1 isolates were repeatedly detected in the Bossembélé district after having been undetected in the country for more than 6 months (Fig. 1). Two additional VDPV2s (CAF-23-023 and CAF-23-169, Fig. 3) with no genetic links with one another or with any other known VDPV2s were detected in February and May 2023 in Sangha-Mbaéré and Nana-Grébizi districts, respectively (Fig. 1 and 2). They were classified as “ambiguous VDPV2s” (aVDPV2), following the GPEI’s terminology used to classify orphan VDPVs, i.e., VDPVs that have no genetic linkages with previous VDPVs (17). Two additional nOPV2 SIAs were implemented in the CAR in June and September 2023 (Table S1). Administrative delays and difficulties in mobilizing resources meant that these campaigns were implemented later than the period recommended by the GPEI. No VDPV2s were detected in the CAR for more than 1 year after October 2023. Overall, 54 VDPV2s were isolated between November 2021 and October 2023 (Table S2), 22 and 16 of which were isolated in stools from AFP and contacts, respectively, and 16 in wastewater (Table 1). Thirty-five isolates with less than six nt differences in the VP1 compared to Sabin 2 were also isolated in Bangui and 12 different health districts (Fig. 2; Table S3) from stools and wastewater samples (Table 1), mainly during the weeks following one of the four nOPV2 SIAs (Fig. 1).

**TABLE 1:** Source of the poliovirus isolates

Category or emergence group	AFP	Contact	Wastewater	Total
NIE-ZAS-1	7	10	3	20
CAF-BNG-2	3	0	12	15
RDC-BUE-1	1	0	0	1
CAF-KEM-1	1	1	0	2
CAF-MOZ-1	2	3	0	5
CAF-BNG-3	6	2	1	9
aVDPV2-n	2	0	0	2
nOPV2-L	18	7	10	35

### Genetic characterization of VDPV2 isolates based on whole genomes

nOPV2 and Sabin 2 genomes are virtually similar to one another, except in the regions that have been modified to stabilize the attenuated phenotype. In particular, the VP1-encoding region cannot be used to distinguish nOPV2 derivatives from Sabin 2 derivatives. Nonetheless, three silent mutations introduced in nOPV2 VP4 and VP2 regions can be used to assess the origin of VDPV2s. Whole-genome sequencing revealed that CAF-MOZ-1 and CAF-BNG-2 emergences stem from the historical Sabin 2 vaccine strain, while CAF-KEM-1 and CAF-BNG-3 emergences and both aVDPV2s (CAF-23-023 and CAF-23-169) were derived from nOPV2; nOPV2-derived VDPV isolates were classified as “VDPV2-n” isolates, following the GPLN’s terminology. All VDPV2-n isolates were subjected to whole-genome sequencing. A subset of Sabin 2-derived VDPVs was also fully sequenced, after selecting representatives of different lineages observed in the VP1-based phylogenetic tree (Fig. 3).

**Fig 4:**
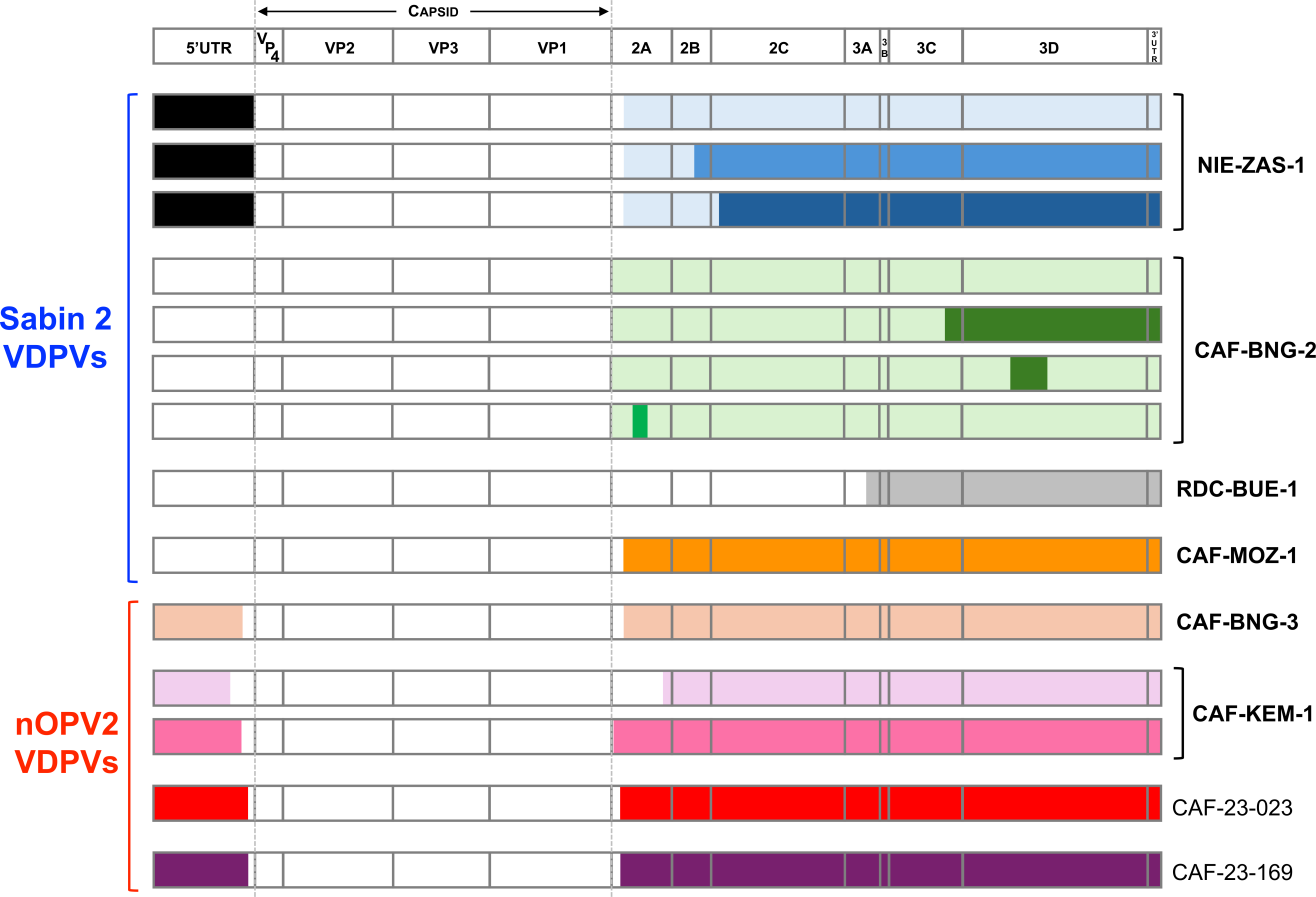
Schematic representation of the genomic patterns of the VDPV2s and VDPV2-ns. The top row shows poliovirus genetic organization, with the main open reading frame flanked by the 5′ and 3′ untranslated regions. Regions from Sabin 2 or nOPV2 are in white, while other regions come from nonpolio enteroviruses. Regions with different colors differ by >3% at the nt level.

All VDPV2s contained sequences from non-polio origin (Tables S4 and Table S5). The members of three emergence groups (CAF-BNG-2, RDC-BUE-1, and CAF-MOZ-1) had a 5′ UTR from Sabin 2, in which the main attenuation determinant (nt position 481) had reverted. The other VDPV2s had a 5′ UTR coming from nonpolio EV-Cs. Multiple recombination patterns were observed downstream of the capsid, with the polio/nonpolio breakpoints mainly located in the 2A region, except for the single representative of RDC-BUE-1 emergence, in which this breakpoint was in the 3A region (Fig. 4; Table S3). Nonpolio sequences found in different emergence groups differ from each other. Furthermore, different recombination patterns were observed within some emergence groups. Thus, all NIE-ZAS-1 members were relatively close to each other from the 5′ UTR through 2A, but three distinct lineages were observed downstream, indicating that additional recombination events occurred in the 2B-3′ UTR part of the genome. Similarly, four different recombination patterns were observed among members of the CAF-BNG-2 emergence: although all were close to each other in most parts of the genome, genetic exchanges were detected in the 2A, 3C, and 3D nonpolio sequences (Fig. 4). The two members of CAF-KEM-1 emergence were very close to each other in the first half of the genome but substantially differed downstream the capsid: first, the polio/nonpolio breakpoint was near the VP1/2A junction in the CAF-23-005CC genome, while it was closer to the 2A/2B junction in the CAF-23-001 genome; second, the nonpolio nonstructural sequences of these two genomes were phylogenetically distinct. By contrast, only one single recombination pattern was observed in each of the CAF-MOZ-1 and CAF-BNG-3 emergences.

A BLAST analysis was conducted on nonstructural genes to identify nonpolio EV-Cs sharing recent common ancestors with the VDPV2s described in this study. No hit with nt similarity >97% was identified, even among EV-Cs sampled in the CAR in 2019–2020 (18).

### Genetic characterization of nOPV2-like isolates based on whole genomes

**Fig 5:**
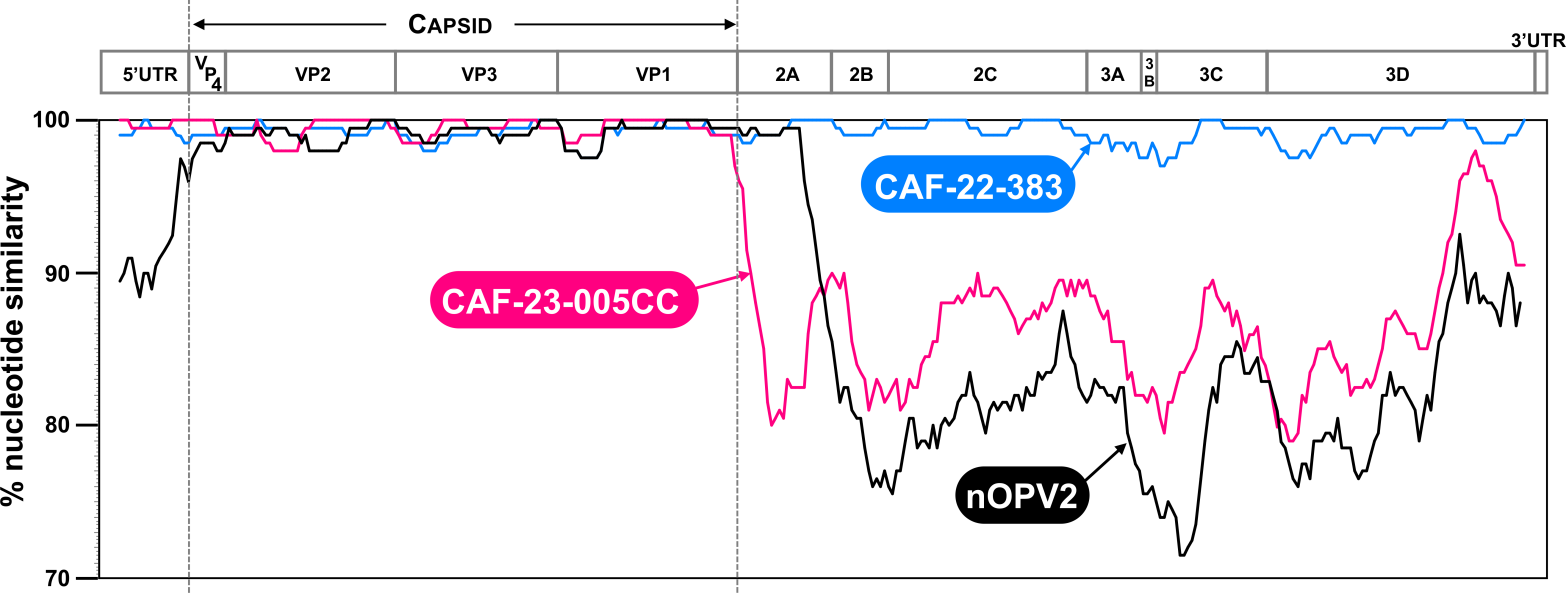
Pairwise comparison of the genome of CAF-23-001 (used as reference) with those of CAF-22-383, CAF-23-005CC, and nOPV2.

All 35 isolates with <6 nt differences in the VP1 compared to Sabin 2 were from nOPV2 and were consequently classified as nOPV2-like (nOPV2-L) isolates (15). All nOPV2-L isolates underwent whole-genome sequencing. Contigs that span virtually all the genome were obtained for all isolates except for one (CAF-23-367), whose first 200 nt were missing because of low coverage in this region; because this virus did not present any programmatic importance, we did not attempt to bridge this gap. All nOPV2-L isolates but two had nonrecombinant genomes. One recombinant isolate (ENV-CAF-22-098-B6) had a single breakpoint, located upstream of the 3C/3D junction (around nt position 5858, according to nOPV2 numbering); the mutated cre located in the 2C was still present in this genome (Table S5). The second recombinant nOPV2-L isolate, CAF-22-383, had a double recombinant genome (Table S5) that was very close (>99% nt identity) in all genomic regions to CAF-23-001, a VDPV2-n of CAF-KEM-1 emergence (Fig. 5). CAF-22-383 and CAF-23-001 were both sampled in the Kemo district, in September 2022 and March 2023, respectively. They featured four and nine nt differences compared to Sabin 2 in the VP1, respectively, of which only one was shared between both isolates.

Of note, no nOPV2-L isolate displayed mutations within the modified domain V, which is the primary attenuation site of nOPV2 (Table 2). Only a few reversions were observed in the other regions that have been modified to engineer nOPV2 (Table 2). Specifically, two nt positions within cre5 exhibited reversions (13 of the 33 isolates carried a change at nt position 123 and a single isolate at nt position 181), and a single nt reversion was observed in the mutated cre region (1 of 34 isolates). A back-mutation of the codon introduced into the 3D polymerase to diminish recombination frequency (Rec1) occurred in one of the 33 nonrecombinant nOPV2-L isolates, whereas the codon engineered to enhance polymerase fidelity (HiFi3) (4) remained unchanged in all nonrecombinant isolates.

**TABLE 2:** Frequency of key mutations found in nOPV2-L isolates

Region	nt position	Mutation
Cre5	123–183	T123C: 13/33^*a*^ A181G: 1/33^*a*^
Domain V	530–595	No mutation
Mutated *cre*	4516–4543	C4543T: 1/34^*b*^
Rec1	6158–6160	Arg38Lys: 1/33^*c*^
Hifi3	6203–6205	Asn53Asp: 0/33^*c*^

^*a*^ This position has a nonpolio origin in CAF-22-383 (double recombinant) and was not sequenced in the CAF-22-367 genome.

^*b*^ This position has a nonpolio origin in CAF-22-383 (double recombinant).

^*c*^ This position has a nonpolio origin in CAF-22-383 (double recombinant) and ENV-CAF-22-098-B6 (single recombinant).

### Evolution of the nOPV2 evolution rate in the capsid region

**Fig 6:**
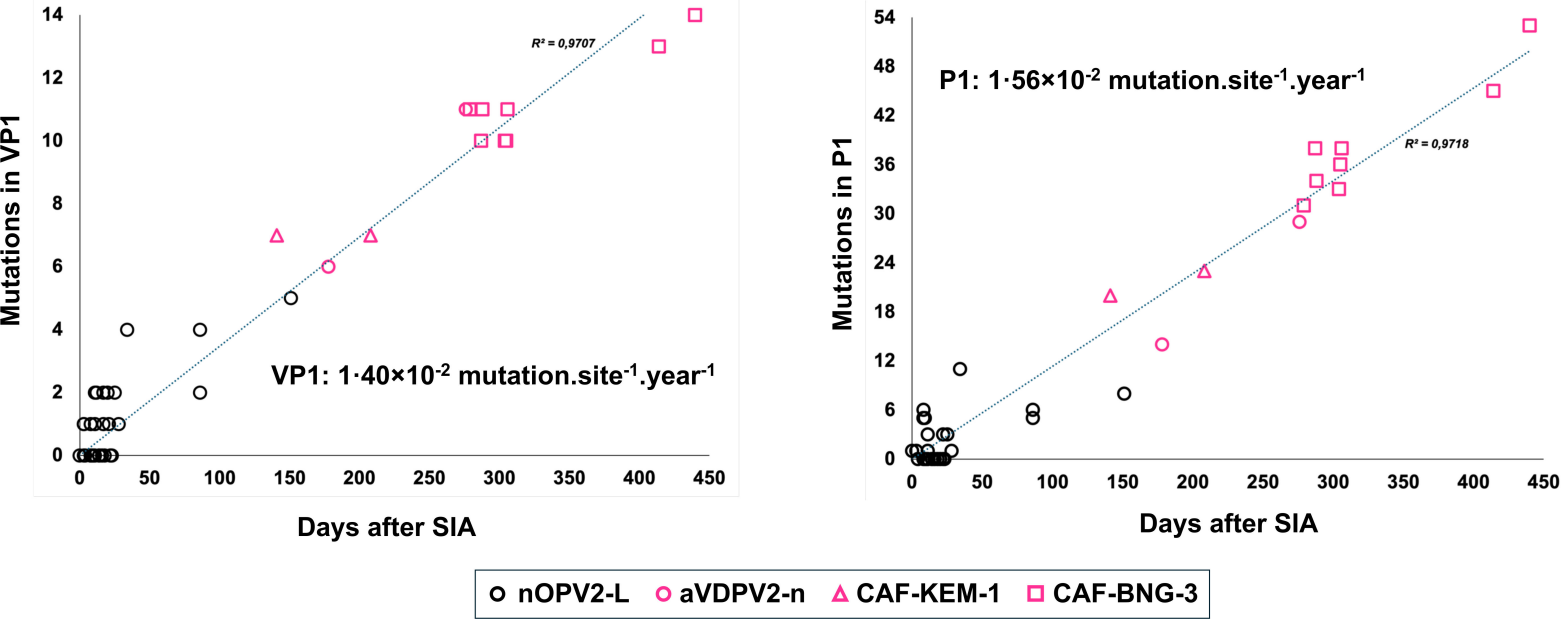
Number of mutations from the nOPV2 vaccine strain in the VP1 (left panel) and P1 (right panel) in nOPV2 derivatives sampled in the Central African Republic as a function of days after supplemental immunization activities (SIAs). The sequence evolution rates estimated by linear regression are indicated in the figure.

To compare the evolution rate of nOPV2 capsid calculated based on our data to evolution rates previously reported for VDPVs, the number of mutations observed in genomic regions encoding VP1 or the whole capsid (P1) of nOPV2 derivatives was plotted as a function of their age, calculated based on the assumption that each isolate was derived from the nOPV2 strain delivered during the latest SIA that took place in the CAR before its detection. The rate of mutations estimated by linear regression was ~1.40 × 10^−2^·site^−1^·year^−1^ (correlation coefficient: 0.9707) in the VP1, and ~1.56 × 10^−2^·site^−1^·year^−1^ (correlation coefficient: 0.9718) in the P1 region (Fig. 6).

## DISCUSSION

This study reports the detection in the CAR of VDPV2s originating from the nOPV2, which was specifically engineered to minimize reversion to a neurovirulent phenotype. The isolates of the emergence group CAF-KEM-1 constituted the first VDPV2-n isolates ever reported to the GPEI. Genetic characterization revealed that all VDPV2-n isolates detected in the CAR arose from two separate recombination events, one upstream and one downstream of the capsid-coding region, which led to the complete loss of attenuation determinants. The CAR remains a high-risk country for poliomyelitis outbreaks due to difficulties in maintaining high vaccine coverage and surveillance in the whole country because of logistical issues and insecurity (19, 20). The vaccine coverage of children with three doses of OPV has been continuously below 50% since 2017 (48% in 2024) (21), and environmental surveillance covers few towns, which impairs the early detection of VDPV emergences or introductions in most parts of the country. This situation explains the concomitant emergences of multiple VDPV2 lineages in the country in 2019, which were unveiled by clinical cases and not by environmental surveillance (7, 8). The country’s situation is even more fragile as it is surrounded by countries that also regularly experience emergences of VDPVs that can be imported by trans-border crossings, as exemplified by the detection of a VDPV2 belonging to the RDC-BUE-1 emergence group. For reasons that remain unclear, this emergence group apparently did not establish sustained transmission chains in the CAR, unless they have simply gone undetected because of sub-optimal surveillance. It is impossible to ascertain whether the other VDPV2s detected in the CAR emerged from vaccine doses delivered in the country or from doses given elsewhere. The last doses of historical Sabin 2 strains used in the CAR were administered in December 2020. Given the mutation rate in the VP1 of roughly 1% per site per year, this timescale is compatible with the emergence of the CAF-BNG-2 and CAF-MOZ-1 isolates from the latest Sabin 2 doses delivered in the CAR. Nevertheless, these strains may have been imported from any country that was still using Sabin 2 at the time as polioviruses disseminate readily owing to asymptomatic infections and prolonged viral shedding that can persist for several weeks.

All VDPV2s, irrespective of whether they were derived from Sabin 2 or nOPV2, featured recombination patterns usually observed in VDPV genomes, with polio/nonpolio breakpoints located near the 5′ UTR/VP4, VP1/2A, 2A/2B, and 2C/3A junctions, known as recombination hotspots (22). As previously observed (7), isolates from the same emergence group differ substantially from each other in the nonpolio regions, thus highlighting the constant genetic mixing induced by recombination among co-circulating EVs. We did not identify in publicly available databases any putative parental strains that could have supplied the nonpolio sequences present in the recombinant VDPV2 and VDPV2-n genomes, a finding that likely reflects the under-reporting of these viruses. In fact, nonpolio EV-Cs are not targeted by clinical surveillance because they are not associated with severe diseases, apart from a variant of coxsackievirus A24 responsible for outbreaks of acute hemorrhagic conjunctivitis. Moreover, nonpolio EV-Cs do not replicate well in the cell lines employed for poliovirus surveillance and are therefore underdetected among the nonpolio enteroviruses that are recovered as a by-product of poliovirus surveillance activities.

Although nOPV2 accumulates less mutations than Sabin 2 because of the Hifi3 mutation introduced in its 3D polymerase (4), this phenotypic property is lost once the genomic region that encodes the polymerase is exchanged with another enterovirus. Therefore, recombinant nOPV2 derivatives probably evolve as Sabin 2 derivatives. The estimated mutation rates of nOPV2 derivatives in VP1 and P1 regions were slightly higher than those previously reported for circulating VDPV2s (1.05–1.20 × 10^−2^·site^−1^·year^−1^ and 1.47 × 10^−2^·site^−1^·year^−1^ in P1 for VDPV2s sampled in Nigeria [23] and in Estonia [24], respectively). The reliability of these estimates may be limited by the number of isolates and the assumption that nOPV2 isolates originate exclusively from viruses administered during the previous SIA in the CAR, whereas some isolates may have been imported from other countries. The absence of mutations within the nOPV2-L modified domain V is consistent with findings from previous clinical trials (25) and subsequent characterization of nOPV2 isolates in Uganda (13) and supports the conclusion that the genetic modifications introduced into nOPV2 effectively prevent phenotypic reversion through genetic drift. However, reversion via recombination with nonpolio EV-Cs remains possible, as proven by the detection of four independent VDPV2-n emergences in the CAR within a few months. The CAR likely provides a favorable ecological context for such recombination events, given the relatively high prevalence of nonpolio EV-Cs in this country (18, 26), as is the case in other Sub-Saharan African countries (27). This is in line with the detection of double recombinant VDPV2-n isolates in Burundi, the Democratic Republic of Congo, Tanzania, Nigeria, Uganda, and Zambia (13, 28, 29).

In accordance with the GPLN algorithm, isolates that differ by fewer than six nt in VP1 from Sabin 2 are generally not sequenced beyond VP1. The cutoff employed to discriminate Sabin 2-like (and now nOPV2-L) isolates from VDPV2s was established empirically from field observations: isolates exhibiting more than six nt differences are presumed to have arisen through human-to-human transmission, thereby indicating individuals with low or absent immunity to polioviruses. Conversely, isolates with fewer than six nt differences are unlikely to have phenotypically reverted, whereas those with six or more nt differences are considered likely to have lost attenuation determinants either by genetic drift (for the historical Sabin 2 strain) or through recombination (for both Sabin 2 and nOPV2). Nonetheless, a gray zone persists, as illustrated by CAF-22-383, an nOPV2-L isolate (<6  nt differences) that had already undergone recombination both upstream and downstream of the capsid-encoding region. The close genetic relationship between this isolate and CAF-23-001 (a VDPV2-n isolate) strongly suggests that both originated from a nOPV2 derivative circulating for only a short period prior to recombination or possibly that recombination occurred directly in the vaccine recipient. Specifically, their high sequence similarity across the genome makes it unlikely that they result from two independent recombination events, and they share only one nt difference compared to nOPV2 in the VP1 region. These observations strongly suggest that their most recent common ancestor was a double recombinant differing from nOPV2 by one nt at most.

A limitation of this study is the quality of the surveillance conducted in the CAR. Logistical and security issues hamper the proper, continuous, high-quality implementation of poliovirus surveillance across the country. Therefore, it is impossible to ascertain that no VDPV2 was circulating in the CAR after October 2023. Consequently, our results do not demonstrate the impact of nOPV2 use on VDPV2 circulation.

nOPV2 represents a clear improvement over the historical Sabin 2 strain. The risk of VDPV2 emergence was estimated to be four-fold lower following nOPV2 use than Sabin 2 use (30). Due to the increased genetic stability of the modified domain V, low vaccine coverage is no longer the sole condition required for VDPV2 emergence when nOPV2 is used. Instead, the presence of nonpolio EV-Cs has become a key factor enabling nOPV2 reversion through recombination. Consequently, identifying whether specific EV-C genetic lineages are particularly prone to generate high-fitness VDPV2s could be critical for mitigating the risk of VDPV2 emergence. Therefore, dedicated investigations of nonpolio EV-Cs aiming to close existing gaps in genetic databases and to assess potential seasonal circulation patterns could inform the design of vaccination strategies tailored to minimize the risk of recombination between nOPV2 and nonpolio EV-Cs, especially in Sub-Saharan Africa. If this strategy fails, further genetic modifications could be introduced into the genomes of the vaccine strains to limit the spread of recombinant VDPVs. Because any genomic segment situated upstream or downstream of the capsid-encoding region may be lost through recombination, such modifications ought to be engineered within the capsid-encoding region itself. Strains with modified capsid-encoding regions were previously developed, for instance, by incorporating numerous silent mutations or by adapting poliovirus strains to relatively low temperatures. Although the resultant viruses were ultimately not selected for the development of nOPV2, the approach of modifying the capsid-coding region remains a viable avenue for future investigation. It could be redeployed to curtail the circulation of recombinant VDPVs in locations where nonpolio EV-C prevalence is high and where sustaining a high level of vaccine coverage proves challenging.

## Data Availability

Poliovirus genetic sequences were submitted to GenBank (accession numbers: PX000236–PX000267, PX000269–PX000317, and PX939681).

## SUPPLEMENTAL MATERIAL

Fig S1 mbio.00669-26-s0001.pdf Locations of the wastewater sampling sites in Central African Republic.

Table S1 mbio.00669-26-s0002.pdf National immunization activities with nOPV2 conducted in CAR in 2022 and 2023.

Table S2 mbio.00669-26-s0003.pdf List of VDPV2 and VDPV2-n isolates.

Table S3 mbio.00669-26-s0004.pdf List of nOPV2-L isolates.

Table S4 mbio.00669-26-s0005.pdf Location of the breakpoints in the VDPV2 genomes.

Table S5 mbio.00669-26-s0006.pdf Location of the breakpoints in the nOPV2-derived recombinant genomes.

ASM does not own the copyrights to Supplemental Material that may be linked to, or accessed through, an article. The authors have granted ASM a non-exclusive, world-wide license to publish the Supplemental Material files. Please contact the corresponding author directly for reuse.
