# Supplementary figures and images for: Emergence of vaccine-derived poliovirus strains from the novel oral polio vaccine in the Central African Republic
Source: mBio. 2026 Apr 23;17(5):e00669-26. doi: 10.1128/mbio.00669-26 (PMC13170175; doi:10.1128/mbio.00669-26)

Supplementary Fig.1. Locations of the wastewater sampling sites in Central African Republic.

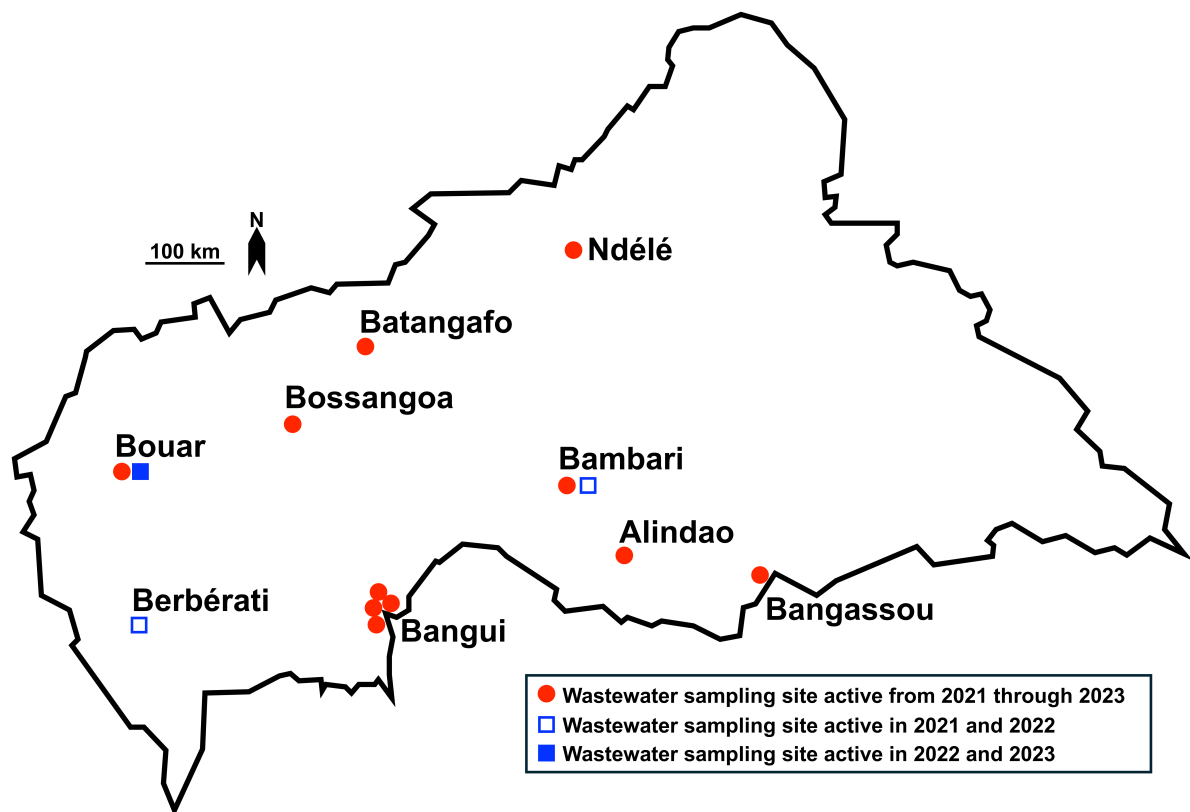

Supplement: Fig S1 — Locations of the wastewater sampling sites in Central African Republic. [file mbio.00669-26-s0001.pdf]
