# Supplementary material for: Emergence of vaccine-derived poliovirus strains from the novel oral polio vaccine in the Central African Republic
Source: mBio. 2026 Apr 23;17(5):e00669-26. doi: 10.1128/mbio.00669-26 (PMC13170175; doi:10.1128/mbio.00669-26)
Supplement: Table S1 — National immunization activities with nOPV2 conducted in CAR in 2022 and 2023. [file mbio.00669-26-s0002.pdf]

Supplementary Table 1. National immunization activities with nOPV2 conducted in CAR in 2022 and 2023.

Period	Target population size	Vaccinated children	Target-population reach (%)
03/06/2022-06/06-2022	1,310,108	1,448,396	111
05/08/2022-22/08/2022	1,310,108	1,611,187	123
02/06/2023-06/06/2023	1,626,634	1,748,271	107
07/09/2023-10/09/2023	1,626,634	1,838,927	113
