# Supplementary material for: Emergence of vaccine-derived poliovirus strains from the novel oral polio vaccine in the Central African Republic
Source: mBio. 2026 Apr 23;17(5):e00669-26. doi: 10.1128/mbio.00669-26 (PMC13170175; doi:10.1128/mbio.00669-26)
Supplement: Table S2 — List of VDPV2 and VDPV2-n isolates. [file mbio.00669-26-s0003.pdf]

Supplementary Table 2. List of VDPV2 and VDPV2-n isolates.

Isolate	Source	District	Date of sampling	GenBank Accession No	Emergence group
ENV-CAF-21-110-B7	Wastewater	Bangui	03/11/2021	PX000306	NIE-ZAS-1
ENV-CAF-22-054-B7	Wastewater	Bangui	04/05/2022	PX000257	NIE-ZAS-1
ENV-CAF-22-055-B3	Wastewater	Bangui	04/05/2022	PX000258	NIE-ZAS-1
CAF-22-437	Stool	Bossangoa	31/12/2022	PX000283	NIE-ZAS-1
CAF-23-027	Stool	Bossembélé	09/02/2023	PX000239	NIE-ZAS-1
CAF-23-063	Stool	Bossembélé	27/02/2023	PX000242	NIE-ZAS-1
CAF-23-107	Stool	Bossembélé	23/03/2023	PX000251	NIE-ZAS-1
CAF-23-122	Stool	Bossangoa	09/04/2023	PX000290	NIE-ZAS-1
CAF-23-056CC	Stool	Bossembélé	16/04/2023	PX000241	NIE-ZAS-1
CAF-23-067CC	Stool	Bossembélé	16/04/2023	PX000244	NIE-ZAS-1
CAF-23-069CC	Stool	Bossembélé	16/04/2023	PX000245	NIE-ZAS-1
CAF-23-078CC	Stool	Bossembélé	18/04/2023	PX000246	NIE-ZAS-1
CAF-23-085CC	Stool	Bossembélé	18/04/2023	PX000247	NIE-ZAS-1
CAF-23-089CC	Stool	Bossembélé	18/04/2023	PX000248	NIE-ZAS-1
CAF-23-092CC	Stool	Bossembélé	18/04/2023	PX000289	NIE-ZAS-1
CAF-23-098CC	Stool	Bossembélé	18/04/2023	PX000250	NIE-ZAS-1
CAF-23-060CC	Stool	Bossembélé	16/04/2023	PX000288	NIE-ZAS-1
CAF-23-066CC	Stool	Bossembélé	16/04/2023	PX000243	NIE-ZAS-1
CAF-23-294	Stool	Carnot- Gadzi	29/07/2023	PX000256	NIE-ZAS-1
CAF-23-334	Stool	Carnot- Gadzi	18/09/2023	PX000303	NIE-ZAS-1
ENV-CAF-22-055-B1	Wastewater	Bangui	04/05/2022	PX000307	CAF-BNG-2
ENV-CAF-22-098-B4	Wastewater	Bangui	29/06/2022	PX000309	CAF-BNG-2
CAF-22-305	Stool	Bangui	27/07/2022	PX000236	CAF-BNG-2
ENV-CAF-22-115-B1	Wastewater	Bangui	27/07/2022	PX000259	CAF-BNG-2
ENV-CAF-22-115-B6	Wastewater	Bangui	27/07/2022	PX000260	CAF-BNG-2
ENV-CAF-22-117-B7	Wastewater	Bangui	26/07/2022	PX000311	CAF-BNG-2
CAF-22-313	Stool	Mbaïki	08/08/2022	PX000275	CAF-BNG-2
CAF-22-355	Stool	Mbaïki	23/08/2022	PX000277	CAF-BNG-2
ENV-CAF-22-155-B1	Wastewater	Bangui	21/09/2022	PX000261	CAF-BNG-2
ENV-CAF-22-155-B2	Wastewater	Bangui	21/09/2022	PX000262	CAF-BNG-2
ENV-CAF-22-155-B7	Wastewater	Bangui	21/09/2022	PX000263	CAF-BNG-2
ENV-CAF-22-191-B3	Wastewater	Bangui	09/11/2022	PX000264	CAF-BNG-2
ENV-CAF-22-191-B5	Wastewater	Bangui	09/11/2022	PX000265	CAF-BNG-2
ENV-CAF-22-191-B6	Wastewater	Bangui	09/11/2022	PX000266	CAF-BNG-2
ENV-CAF-22-199	Wastewater	Bangui	23/11/2022	PX000267	CAF-BNG-2
CAF-22-421	Stool	Bangassou	26/11/2022	PX000282	RDC-BUE-1
CAF-23-019	Stool	Mobaye-Zangba	28/01/2023	PX000286	CAF-MOZ-1
CAF-23-043	Stool	Mobaye-Zangba	18/02/2023	PX000240	CAF-MOZ-1

CAF-23-020-C1	Stool	Mobaye-Zangba	19/02/2023	PX000237	CAF-MOZ-1
CAF-23-020-C2	Stool	Mobaye-Zangba	19/02/2023	PX000238	CAF-MOZ-1
CAF-23-171CC	Stool	Mobaye-Zangba	26/04/2023	PX000253	CAF-MOZ-1
ENV-CAF-23-084	Wastewater	Bangui	19/05/2023	PX000314	CAF-BNG-3
CAF-23-226	Stool	Alindao-Mingala	13/06/2023	PX000299	CAF-BNG-3
CAF-23-112-C1	Stool	Alindao-Mingala	14/06/2023	PX000252	CAF-BNG-3
CAF-23-190	Stool	Bangui	15/06/2023	PX000255	CAF-BNG-3
CAF-23-188	Stool	Bambari	27/05/2023	PX000293	CAF-BNG-3
CAF-23-189	Stool	Bambari	28/05/2023	PX000254	CAF-BNG-3
CAF-23-093-C3	Stool	Bambari	28/05/2023	PX000249	CAF-BNG-3
CAF-23-351	Stool	Alindao-Mingala	01/10/2023	PX000304	CAF-BNG-3
CAF-23-370	Stool	Bambari	27/10/2023	PX000305	CAF-BNG-3
CAF-23-001	Stool	Kémo	01/01/2023	PX000284	CAF-KEM-1
CAF-23-005CC	Stool	Kémo	09/03/2023	PX000283	CAF-KEM-1
CAF-23-023	Stool	Sangha-Mbaéré	07/02/2023	PX000287	VDPV2-n
CAF-23-169	Stool	Nana-Grézibi	16/05/2023	PX000292	VDPV2-n
