# Supplementary material for: Emergence of vaccine-derived poliovirus strains from the novel oral polio vaccine in the Central African Republic
Source: mBio. 2026 Apr 23;17(5):e00669-26. doi: 10.1128/mbio.00669-26 (PMC13170175; doi:10.1128/mbio.00669-26)
Supplement: Table S3 — List of nOPV2-L isolates. [file mbio.00669-26-s0004.pdf]

Supplementary Table 3. List of nOPV2-L isolates.

Isolate	Source	District	Date of sampling	GenBank Accession No
CAF-22-117-C1	Stool	Alindao-Mingala	14/06/2022	PX000270
ENV-CAF-22-085	Wastewater	Bangui	15/06/2022	PX000308
CAF-22-260	Stool	Bangassou	18/06/2022	PX000274
CAF-22-128-C3	Stool	Bangassou	18/06/2022	PX000273
CAF-22-127-C1	Stool	Alindao-Mingala	26/06/2022	PX000271
CAF-22-127-C2	Stool	Alindao-Mingala	26/06/2022	PX000272
ENV-CAF-22-098-B6	Wastewater	Bangui	29/06/2022	PX000310
CAF-22-012-CC	Stool	Bangui	02/07/2022	PX000269
CAF-22-339	Stool	Bossembélé	17/08/2022	PX000276
CAF-22-359	Stool	Haute-Kotto	24/08/2022	PX000278
ENV-CAF-22-134-B6	Wastewater	Bangui	24/08/2022	PX000312
CAF-22-360	Stool	Haute-Kotto	25/08/2022	*
CAF-22-367	Stool	Bouca	29/08/2022	PX000279
CAF-22-371	Stool	Bangassou	02/09/2022	PX000280
CAF-22-372	Stool	Bangassou	03/09/2022	§
CAF-22-383	Stool	Kémo	16/09/2022	PX000281
ENV-CAF-23-004-B8	Wastewater	Bangui	11/01/2023	PX000313
ENV-CAF-23-096-B6	Wastewater	Bangui	07/06/2023	PX000315
ENV-CAF-23-096-B7	Wastewater	Bangui	07/06/2023	э
ENV-CAF-23-096-B8	Wastewater	Bangui	07/06/2023	э
CAF-23-220	Stool	Bangui	12/06/2023	PX000295
CAF-23-212	Stool	Bossembélé	12/06/2023	PX000294
CAF-23-222	Stool	Bégoua	12/06/2023	PX000296
CAF-23-225	Stool	Kouango-Grimari	12/06/2023	PX000298
CAF-23-221	Stool	Bangui	13/06/2023	†
ENV-CAF-23-103-B8	Wastewater	Bangui	21/06/2023	‡
CAF-23-223	Stool	Bégoua	13/06/2023	PX000297
ENV-CAF-23-103-B6	Wastewater	Bangui	21/06/2023	PX000316
ENV-CAF-23-105-B6	Wastewater	Bouar-Baoro	22/06/2023	PX000317
CAF-23-250	Stool	Kembe-Satema	27/06/2023	PX000300
CAF-23-251	Stool	Kembé-Satéma	28/06/2023	&
CAF-23-324	Stool	Ouango-Gambo	29/08/2023	PX000301
CAF-23-161-C1	Stool	Ouango-Gambo	29/08/2023	PX939681
CAF-23-157-C1	Stool	Bégoua	07/09/2023	PX000291
CAF-23-332	Stool	Mobaye-Zangba	16/09/2023	PX000302

* Sequence similar to that of CAF-22-359 (same donor), not submitted to GenBank.
§ Sequence similar to that of CAF-22-370 (same donor), not submitted to GenBank.
э Sequence similar to that of ENV-CAF-23-096-B6 (same wastewater sample), not submitted to GenBank.
† Sequence similar to that of CAF-22-220 (same donor), not submitted to GenBank.
‡ Sequence similar to that of ENV-CAF-23-103-B6 (same wastewater sample), not submitted to GenBank.
& Sequence similar to that of CAF-22-250 (same donor), not submitted to GenBank.
