# Supplementary material for: Emergence of vaccine-derived poliovirus strains from the novel oral polio vaccine in the Central African Republic
Source: mBio. 2026 Apr 23;17(5):e00669-26. doi: 10.1128/mbio.00669-26 (PMC13170175; doi:10.1128/mbio.00669-26)
Supplement: Table S4 — Location of the breakpoints in the VDPV2 genomes. [file mbio.00669-26-s0005.pdf]

Supplementary Table 4. Location of the breakpoints in the VDPV2 genomes. The approximate locations of the breakpoints between the Sabin 2 vaccine strains and unidentified non-polio enteroviruses were determined through visual inspection of pairwise alignments.

Isolate	Breakpoint upstream the capsid*	Breakpoint downstream the capsid*	GenBank Accession No
NIE-ZAS-1			
ENV-CAF-21-110-B7	525	3497	PX000306
CAF-22-437	740	3500	PX000283
CAF-23-122	739	3499	PX000290
CAF-23-092CC	743	3503	PX000289
CAF-23-060CC	740	3500	PX000288
CAF-23-334	741	3501	PX000303
CAF-BNG-2			
ENV-CAF-22-055-B1	None	3387	PX000307
ENV-CAF-22-098-B4	None	3387	PX000309
ENV-CAF-22-117-B7	None	3387	PX000311
CAF-22-313	None	3387	PX000275
CAF-22-355	None	3387	PX000277
RDC-BUE-1			
CAF-22-421	None	5307	PX000282
CAF-MOZ-1			
CAF-23-019	None	3459	PX000286
CAF-23-020-C1	None	3459	PX000237
CAF-23-171CC	None	3459	PX000253

* Numbering according to the genome of the Sabin 2 vaccine strain (GenBank accession number AY184220).
