# Supplementary material for: Emergence of vaccine-derived poliovirus strains from the novel oral polio vaccine in the Central African Republic
Source: mBio. 2026 Apr 23;17(5):e00669-26. doi: 10.1128/mbio.00669-26 (PMC13170175; doi:10.1128/mbio.00669-26)
Supplement: Table S5 — Location of the breakpoints in the nOPV2-derived recombinant genomes. [file mbio.00669-26-s0006.pdf]

Supplementary Table 5. Location of the breakpoints in the nOPV2-derived recombinant genomes. The approximate locations of the breakpoints between the nOPV2 vaccine strains and unidentified non-polio enteroviruses were determined through visual inspection of pairwise alignments.

Isolate	Breakpoint upstream the capsid*	Breakpoint downstream the capsid*	Engineered elements§			GenBank Accession No
			5'UTR		2C	
			Mutated domain V	Relocated cre	Mutated cre	
CAF-BNG-3						
ENV-CAF-23-084	641	3565	✗	✗	✗	PX000314
CAF-23-226	639	3563	✗	✗	✗	PX000299
CAF-23-112-C1	640	3564	✗	✗	✗	PX000252
CAF-23-190	640	3564	✗	✗	✗	PX000255
CAF-23-188	640	3563	✗	✗	✗	PX000293
CAF-23-189	639	3563	✗	✗	✗	PX000254
CAF-23-093-C3	640	3564	✗	✗	✗	PX000249
CAF-23-351	639	3563	✗	✗	✗	PX000304
CAF-23-370	641	3563	✗	✗	✗	PX000305
CAF-KEM-1						
CAF-23-001	673	3837	✗	✗	✗	PX000284
CAF-23-005CC	673	3498	✗	✗	✗	PX000283
Ambiguous VDPV2-n						
CAF-23-023	719	3451	✗	✗	✗	PX000287
CAF-23-169	598	3877	✗	✗	✗	PX000292
nOPV2-L						
ENV-CAF-22-098-B6	None	5842	✓	✓	✓	PX000310
CAF-22-383	673	3838	✗	✗	✗	PX000281

* Numbering according to the genome of the nOPV2 vaccine strain (GenBank accession number MZ245455).

§ ✓ Still present; ✗ Lost through recombination.
